# The Acute Effect of Foam Rolling on Eccentrically-Induced Muscle Damage

**DOI:** 10.3390/ijerph18010075

**Published:** 2020-12-24

**Authors:** Masatoshi Nakamura, Koki Yasaka, Ryosuke Kiyono, Remi Onuma, Kaoru Yahata, Shigeru Sato, Andreas Konrad

**Affiliations:** 1 Department of Physical Therapy, Faculty of Rehabilitation, Niigata University of Health and Welfare, 1398 Shimami-cho, Kita-ku, Niigata City, Niigata 950-3198, Japan; hpa17114@nuhw.ac.jp (K.Y.); hpa17024@nuhw.ac.jp (R.O.); 2 Institute for Human Movement and Medical Sciences, Niigata University of Health and Welfare, 1398 Shimami-cho, Kita-ku, Niigata City, Niigata 950-3198, Japan; hpm19005@nuhw.ac.jp (R.K.); hpm20011@nuhw.ac.jp (K.Y.); hpm19006@nuhw.ac.jp (S.S.); 3 Institute of Sport Science, University of Graz, Mozartgasse 14, 8010 Graz, Austria; andreas.konrad@uni-graz.at

**Keywords:** muscle soreness, muscle strength, range of motion, knee extensor

## Abstract

Previous studies have shown significant improvement in muscle soreness and muscle function loss after 300-s foam rolling intervention two days after intense exercise. However, this duration is assumed to be too long, so investigating the effect of short-term duration foam rolling intervention on an eccentrically-damaged muscle is needed. This study aimed to eccentrically induce muscle damage in the leg extensors, and to detect the acute effect of 90-s foam rolling on muscle soreness and muscle function of the quadriceps muscle. We enrolled 17 healthy and nonathlete male volunteers. They performed a bout of eccentric exercise of the knee extensors with the dominant leg and received 90-s foam rolling intervention of the quadriceps two days after the eccentric exercise. The dependent variables were measured before the eccentric exercise (baseline), and before (preintervention) and after foam rolling intervention (postintervention), two days after the eccentric exercise. The results show that the preintervention muscle soreness and muscle strength values were significantly increased, compared with the baseline values, whereas the postintervention values were significantly decreased, compared with the preintervention values. Furthermore, 90-s of foam rolling intervention could improve muscle soreness and muscle function loss.

## 1. Introduction

Compared with resistance training emphasizing concentric contraction, it is well known that resistance training emphasizing eccentric contraction (ECC) allows for greater increases in muscle strength and muscle volume [1,2]. However, previous studies have shown the negative aspects, including delayed-onset muscle soreness (DOMS), muscle function loss (muscle strength or athletic performance), decrease in range of motion (ROM), and increase in muscle-tendon stiffness after performing ECC [3,4,5,6,7]. These muscle damage symptoms peaked in two days and remained for about one week [4,5]. Since DOMS and muscle function loss impair the individual’s willingness to exercise, and inhibit the continuation of exercise for a certain period, it is necessary to establish effective intervention modalities to prevent or treat DOMS and muscle function loss after ECC [8].

In previous studies, the effective intervention modalities to prevent DOMS and muscle function loss were investigated, and a systematic review concluded that active recovery, massage, compression garments, immersion, contrast water therapy, and cryotherapy induced a small-to-large decrease in the magnitude of DOMS and muscle damage. However, there was no significant decrease in the effect of stretching on DOMS [8]. Furthermore, it is well known that a bout of ECC exercise confers protection against DOMS and muscle damage by ECC exercise following a subsequent bout of ECC exercise via the repeated-bout effect [9,10]. Conversely, to the best of our knowledge, the acute effect of some interventions on damaged muscle caused by ECC exercise has been investigated in a few studies. One study investigated the effect of static stretching on eccentrically-damaged muscle two days after ECC exercise and showed that there was an improvement in muscle soreness, ROM, and muscle-tendon stiffness after a static stretching intervention [7]. Additionally, the other studies showed that there was an improvement in muscle soreness and ROM after static stretching and hold–relax stretching interventions [11,12]. These results revealed that stretching intervention might not be effective in preventing DOMS, whereas a stretching intervention could be effective in improving muscle soreness and muscle function in eccentrically-damaged muscle.

Furthermore, many researchers have focused on the foam rolling effect using a foam roller, roller massage bars/sticks, or a ball. The foam rolling effects on muscle strength and athletic performance have not reached consensus [13,14,15,16,17,18]; however, previous studies have shown that a foam rolling intervention increased ROM and pain threshold [16,18,19]. Although Behm and Wilke pointed out that the evidence supporting the fact that the primary mechanisms underlying foam rolling are the release of myofascial restrictions is insufficient [20], foam rolling intervention could be useful in sports and rehabilitation settings, because of its increment effect on ROM and pain threshold. Regarding the recovery effects after foam rolling, previous studies have shown that foam rolling intervention aids in the recovery from muscle damage immediately after 24, 48, and 72 h after ECC exercise [21,22]. Moreover, Romero-Moraleda and colleagues investigated the effect of 300-s foam rolling on the damaged muscle two days after exercise, in which the symptoms of DOMS and muscle function loss peaked, and showed significant improvement in DOMS and function loss [23,24]. However, after specific ECC exercise studies, it was shown that the acute effect of foam rolling on DOMS is limited. Furthermore, Romero-Moraleda and colleagues used the 300-s foam rolling intervention duration, and showed that it is too long for clinical application in sports and rehabilitation settings. Hence, investigating the effect of a short-term duration of foam rolling intervention in the clinical settings is needed. One previous systematic review concluded that a reduction in pain/soreness could be achieved by >90-s foam rolling [25], and we believed that to change the DOMS and muscle function loss, >90-s foam rolling duration is required. This study aimed to eccentrically induce muscle damage of the leg extensors and to investigate the acute effect of 90-s foam rolling intervention on muscle soreness and loss of muscle function of the quadriceps muscle.

## 2. Materials and Methods

### 2.1. Participants

A total of 17 healthy and sedentary male volunteers (mean ± standard deviation (SD): age 21.1 ± 0.5 years; height, 170.9 ± 5.9 cm; weight, 61.1 ± 6.2 kg), who had not performed habitual exercise activities at least for the past six months before the measurements, were enrolled in this study. We excluded participants who had a history of neuromuscular disease or musculoskeletal injury on the lower extremity. All participants had not been involved in any regular resistance training or flexibility training. Previous studies have revealed that the repeated muscle contractions—especially ECC—could attenuate the muscle soreness and muscle function loss: the so-called “repeated-bout effect” [9,10]. Therefore, we included nontrained male participants in this study. All participants provided written informed consent. The study was approved by the Ethics Committee (#18220) and complies with the requirements of the Declaration of Helsinki.

### 2.2. Experimental Protocol

**Figure 1:**
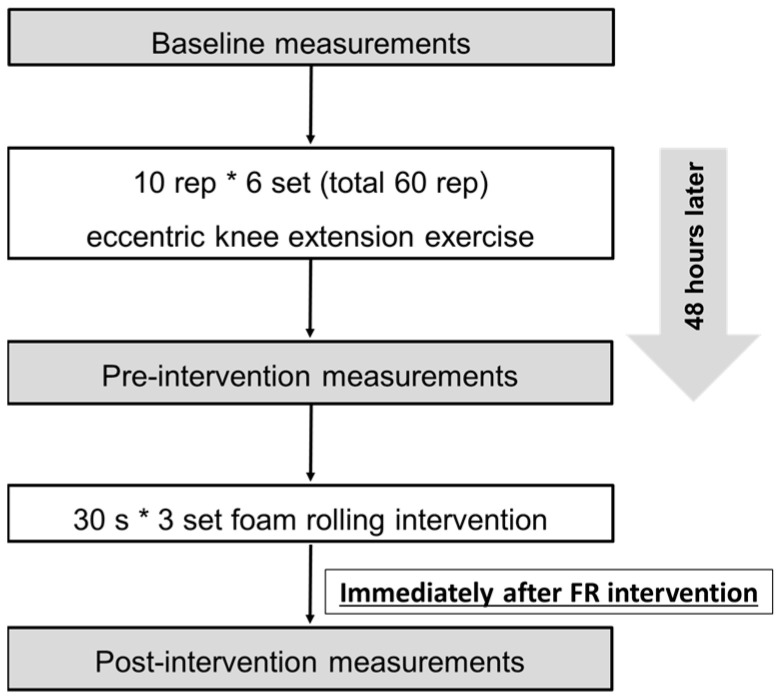
Experimental flowchart.

The participants performed a bout of eccentric exercise of the knee extensors with the dominant leg (preferred leg for kicking a ball), as described in the following (Figure 1), and received 90-s foam rolling intervention (30 s × three sets) of the quadriceps two days after the eccentric exercise [9,10,11]. The dependent variables included muscle soreness at contraction, palpation, and stretching, maximum voluntary isometric contraction (MVC-ISO) torque and maximum voluntary concentric contraction (MVC-CON) torque of knee extensors, and range of motion (ROM) of passive knee flexion. These variables were measured before the eccentric exercise (baseline), and before (preintervention) and after (postintervention) foam rolling intervention two days after the eccentric exercise. Immediately after the foam rolling intervention, we performed the postintervention measurements. All measurements were taken at the same time of the day for each participant between days. Furthermore, the participants practiced the foam rolling intervention on the opposite side (nondominant leg) before baseline and preintervention measurements. Additionally, the participants became familiarized with all measurements and ECC exercises before baseline measurement in the measurement leg (dominant leg).

### 2.3. Eccentric Exercise

On an isokinetic dynamometer (Biodex System 3.0, Biodex Medical Systems Inc., Shirley, NY, USA), all participants performed six sets out of 10 maximal ECC of the unilateral knee extensors (dominant leg) [11,12]. Participants were seated in the dynamometer chair at an 80° hip flexion angle, with adjusted Velcro straps fixed over the trunk, pelvis, and thigh of the exercised limb. The exercised limb knee joint was aligned with the axis of rotation of the dynamometer. According to the previous studies [11,12], the participants were instructed to perform the maximal ECC from a slightly flexed position (20°) to a flexed position (110°) at an angular velocity of 60°/s. After each ECC, the lever arm passively returned the knee joint to the starting position at 10°/s, which gave a 9-s rest between contractions. Each set was repeated 10 times, and a 100-s rest was given between sets to complete the six sets. To generate maximum force, the participants received strong verbal encouragement during each ECC.

### 2.4. Foam Rolling Intervention

**Figure 2:**
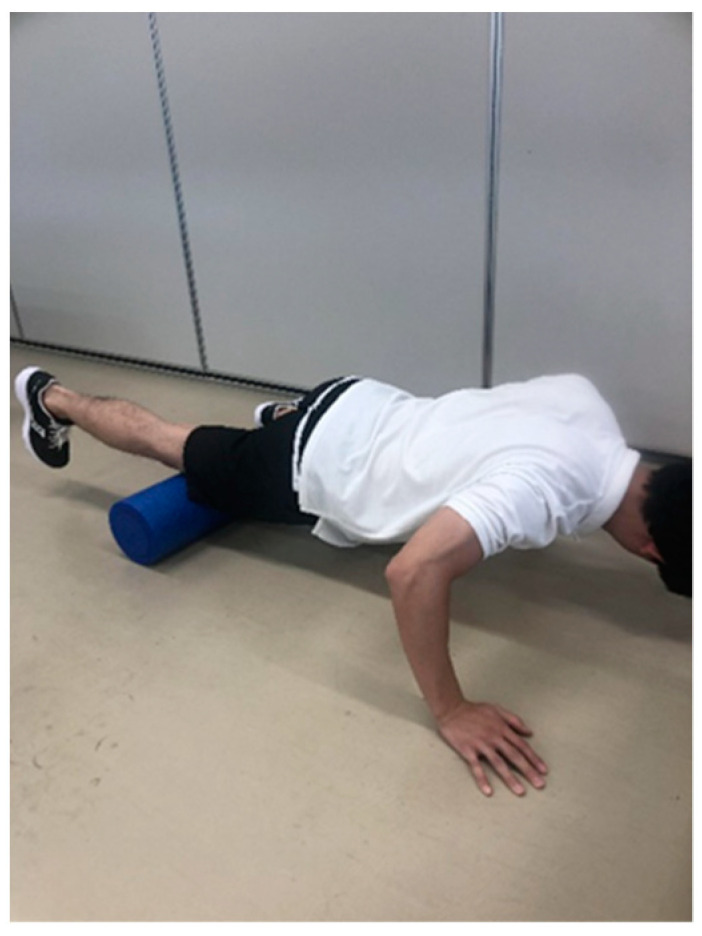
Foam rolling technique.

A foam roller (Gold’s Gym 18 Foam Roller, Logan, UT, USA), with a total diameter of 12.7 cm consisting of a 5-mm thick hollow plastic core covered with a 12-mm layer of dense foam, was used to perform the foam rolling [26]. The subjects were instructed to perform three sets of 30-s foam rolling intervention with 30-s rest between each set. The participants were instructed to be in the plank position with the foam roller at the most proximal portion of the quadriceps of the dominant leg only (Figure 2). This study defined one cycle of foam rolling intervention as one distal rolling plus one subsequent proximal rolling movement, whereas the frequency was defined as 30 cycles per 1 min using a metronome (Smart Metronome; Tomohiro Ihara, Japan). In detail, 15 cycles for each set were completed. The foam rolling intervention was performed between the top of the patella and the anterior superior iliac spine under the direct supervision of investigators. Based on a previous study [27], the pressure was subjectively controlled with a target numerical rating scale rating of 7/10 (0 represents no discomfort and 10 represents maximal discomfort) during the intervention. Additionally, the participants were instructed to control the pressure with a target numerical rating scale rating of 7/10 before each set.

### 2.5. MVC-ISO and MVC-CON

MVC-ISO was measured at two different angles, such as 20° and 70° knee angles, with the same setup as the eccentric exercise using the dynamometer after gravity correction [28]. The participants were instructed to perform maximal contraction for 5 s at each angle two times with 60-s rest between trials, and the average value was adopted for further analysis.

MVC-CON was measured at the angular velocity of 60°/s for the ROM of 70° (20–90° knee angles) for five continuous maximal voluntary concentric contractions, for both directions [11,12]. The highest value among the five trials was adopted for further analysis. During all tests, verbal encouragement was provided consistently.

### 2.6. Knee Flexion ROM

Each participant was placed in a side-lying position on a massage bed, and the hip and knee of the nondominant leg were flexed at 90° to prevent the movement of the pelvis during ROM measurements. The investigator brought the dominant leg to full knee flexion with the hip joint in a neutral position. A goniometer was used to measure the knee flexion ROM twice, and the average value was used for further analysis [11,12].

### 2.7. Muscle Soreness

Using a visual analog scale that had a 100-mm continuous line with “not sore at all” on one side (0 mm) and “very, very sore” on the other side (100 mm), the magnitude of knee extensor muscle soreness was assessed by muscle contraction, stretching, and palpation [9,29]. Muscle soreness at contraction was assessed at both MVC-ISO and MVC-CON, and the average value was adopted for further analysis. For muscle soreness during palpation, participants laid supine on a massage bed, and the investigator palpated the proximal, middle, and distal points of the vastus medialis, vastus lateralis, and rectus femoris [28]. The average value of the knee extensor palpation points was used for further analysis. As for muscle soreness during stretching, muscle soreness during ROM measurement was measured twice, and the average value was used for further analysis.

### 2.8. Test–Retest Reliability of the Measurements

Using seven different healthy males other than those in this study (age, 21.3 ± 0.7 years; height, 173.2 ± 5.9 cm; weight, 62.8 ± 7.3 kg), the test–retest reliability of the measurement for MVIC-ISO, MVIC-CON, ROM, and muscle soreness at contraction, stretching, and palpation was determined by coefficient variation (CV) and intraclass correlation coefficient (ICC), with 5-min rest interval between the two measures in damaged muscle after the same ECC exercise protocol. The CV of the measurements for MVIC-ISO, MVIC-CON, ROM, and muscle soreness at contraction, stretching, and palpation were 4.1% ± 4.1%, 9.2% ± 4.8%, 1.2% ± 0.8%, 10.1% ± 4.5%, 9.9% ± 4.5%, and 8.5% ± 4.2%, respectively, and the ICC for the measurements were 0.98, 0.80, 0.91, 0.96, 0.97, and 0.88, respectively.

### 2.9. Statistical Analysis

SPSS (version 24.0; SPSS Japan Inc., Tokyo, Japan) was used for statistical analysis. By analyzing the standardized residuals using a Shapiro–Wilk test, data were assessed for assumptions of normality. One-way repeated analysis of variance was used to assess significant differences in all variables. When a significant effect was found, the Bonferroni post hoc test was used to determine the differences between measurements taken at baseline, preintervention, and postintervention. Additionally, the effect size (d) was calculated as differences in the mean value divided by the pooled SD [30].

The relationship between changes from baseline to preintervention and from pre- to postintervention in muscle soreness during muscle contraction, stretching, and palpation was quantified using Pearson’s product-moment correlation coefficient. Moreover, it was also used to quantify the relationship between relative changes (%) from baseline to preintervention and from pre- to postintervention in MVC-ISO, MVC-CON, and ROM. Data are presented as mean ± SD.

## 3. Results

Table 1 presents all variables in all groups. The one-way analysis of variance indicated the main effects for all variables. As a result of the post hoc test, muscle soreness at contraction, stretching, and palpation values was significantly increased at preintervention, compared with the baseline values (*p* < 0.01 and d = 1.19; *p* = 0.012 and d = 0.69, *p* < 0.01 and d = 1.57, respectively), whereas postintervention values were significantly decreased, compared with the preintervention values (*p* < 0.01 and d = 0.63; *p* < 0.01 and d = 1.02; *p* < 0.01 and d = 1.27, respectively).

Furthermore, the preintervention MVC-ISO and MVC-CON values were significantly decreased, compared with the baseline values (*p* < 0.01 and d = 1.75; *p* < 0.01 and d = 2.38, respectively), whereas the postintervention values were significantly increased, compared with the preintervention values (*p* < 0.01 and d = 0.45; *p* < 0.01 and d = 0.58, respectively). In addition, the postintervention MVC-ISO and MVC-CON values were significantly lower than the baseline values (*p* < 0.01 and d = 0.74; *p* < 0.01 and d = 0.85, respectively). Similarly, the preintervention ROM values were significantly decreased, compared with the baseline value (*p* < 0.01 and d = 0.75), whereas the postintervention ROM value was significantly increased, compared with the baseline and preintervention values (*p* < 0.01 and d = 0.27; *p* < 0.05 and d = 1.41, respectively).

**Figure 3:**
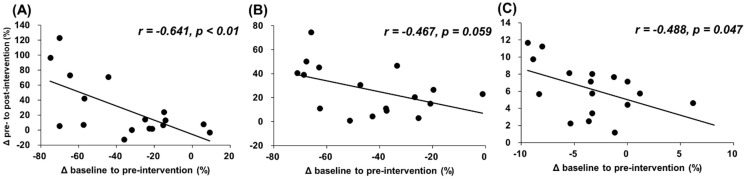
Relationships (Pearson *r* and *p* values) between changes from baseline to preintervention and from pre- to postintervention in muscle soreness at muscle contraction (**A**), stretching (**B**), and palpation (**C**).

**Figure 4:**
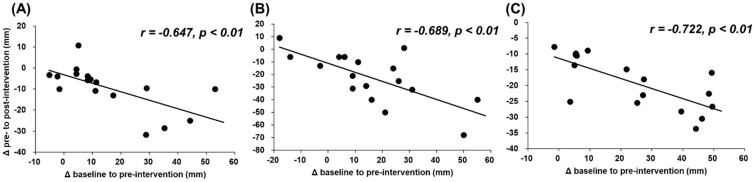
Relationships (Pearson r and p values) between relative changes (%) from baseline to preintervention and from pre- to postintervention in maximal voluntary isometric contraction (MVC-ISO) torque (**A**), maximum voluntary concentric contraction (MVC-CON) torque (**B**) of knee extensors, and range of motion (ROM) of passive knee flexion (**C**).

Figure 3 and Figure 4 show the associations between changes from baseline to preintervention and from pre- to postintervention. The results show that there were significant negative associations between changes (mm) in muscle soreness at muscle contraction, stretching, and palpation (*r* = −0.722 and *p* < 0.01; *r* = −0.689 and *p* < 0.01; *r* = −0.647 and *p* < 0.01, respectively) and relative changes (%) in MVC-ISO and ROM (*r* = −0.641 and *p* < 0.01; *r* = −0.467 and *p* = 0.059; *r* = −0.488 and *p* = 0.047, respectively).

## 4. Discussion

This study investigated 90-s (30 s × three sets) foam rolling intervention on DOMS and muscle function loss 48 h after ECC exercise. The results revealed the following points: (1) The muscle strength loss was recovered after foam rolling intervention, whereas the postintervention values were still lower than the baseline values (MVC-ISO: −25.7%, MVC-CON: −31.9%). (2) After foam rolling intervention, DOMS was recovered, which was similar to the baseline value. (3) After foam rolling, the loss of flexibility was recovered, which was higher than the baseline value. (4) Lastly, the abovementioned foam rolling effect was significantly greater in the participants with higher DOMS and function loss. Romero-Moraleda and colleagues showed that 300-s foam rolling intervention improved DOMS and muscle function loss after intense exercises [23,24]. To the best of our knowledge, this is the first study to investigate the effect of 90-s foam rolling intervention on the damaged muscle that could be applied in the sports and rehabilitation settings.

Our results support and expand the previous works investigating the effect of 300-s foam rolling [23,24]. Interestingly, there is a dose–response relationship between foam rolling duration and foam rolling intervention effect. Moreover, Hughes and Ramer, in their systematic review (2019), showed that >90-s foam rolling intervention could relieve pain/soreness [25]. In this study, DOMS was similarly improved in muscles with eccentrically-induced muscle damage after 90-s foam rolling intervention. The proposed global pain modulatory by foam rolling intervention might be involved with the gate control theory of pain, diffuse noxious inhibitory control, or parasympathetic nervous system alteration [20]. Although the mechanism underlying pain modulatory by foam rolling intervention has been unclear, in this study, 90-s foam rolling intervention could cause pain modulatory and reduce the muscle soreness in eccentrically-damaged muscle.

The findings showed that both MVC-ISO and MVC-CON were improved by foam rolling intervention, which was consistent with the previous studies [23,24]. However, although no significant difference in muscle soreness was observed between baseline and postintervention, there were still significant decrements in MVC-ISO and MVC-CON at postintervention, rather than at baseline, which showed that there could not be a full recovery at baseline. The discrepancy between changes in muscle soreness and function loss after foam rolling intervention has been unclear. Previous studies have shown that >90-s foam rolling could achieve a short-term reduction in pain/soreness [25], but the effects of foam rolling intervention on muscle strength and athletic performance have been debated [13,14,15,16,17,18]. Therefore, although muscle soreness improvement during muscle contraction by the foam rolling intervention improved both MVC-ISO and MVC-CON, the improvement effects on both MVC-ISO and MVC-CON might not be lower than that in muscle soreness.

Interestingly, Matsuo and colleagues (2015) investigated the effect of 300-s static stretching on eccentrically-damaged muscle and revealed that static stretching intervention improved DOMS, whereas there were no significant changes in muscle strength [7]. Generally, muscle strength and athletic performance decrease immediately after static stretching. Therefore, the previous study stated that the possibility of decreased pain sensation counteracted the force loss of eccentrically-damaged muscle after a static stretching intervention [7]. Conversely, although the effects of foam rolling intervention on muscle strength and athletic performance have been debated [13,14,15,16,17,18], to the best of our knowledge, a study showing the decrement effect after foam rolling intervention has not been conducted yet. Therefore, foam rolling intervention for eccentrically-damaged muscle could improve muscle strength loss by improving DOMS. Altogether, the foam rolling intervention could be an effective recovery tool superior to static stretching as an intervention modality for eccentrically-damaged muscle in the sports and rehabilitation settings. In future studies, a comparison of the effects of foam rolling and static stretching on the eccentrically-damaged muscles is needed.

This study revealed that there was a significant negative correlation between the deterioration of DOMS and muscle function loss by ECC exercise and the improvement effect of foam rolling intervention (Figure 3 and Figure 4). These results showed that the subjects with greater muscle soreness or decreased muscle function loss after the ECC exercise were shown to improve greatly after foam rolling intervention. As mentioned above, ECC exercise can be expected to have a great muscle strengthening/muscular hypertrophy effect; however, it has the problem of causing muscle soreness and prolonged muscle function loss. From these study results, since foam rolling intervention on eccentrically-damaged muscle could improve and attenuate the DOMS and muscle function loss, the foam roller can be used as a recovery tool for eccentrically-damaged muscle, controlling the muscle soreness and muscle function loss in sport and rehabilitation settings, especially for the subjects with greater muscle soreness or decreased muscle function loss after the ECC exercise.

There are some limitations to the present study. First, there was no control group (no foam rolling intervention group) in this study. However, the high reliabilities for all measurements were confirmed in the eccentrically-induced damaged muscles. Therefore, there was a possibility that the changes in muscle soreness and muscle function loss could be involved by 90-s foam rolling intervention. Second, since only the acute effect of 90-s foam rolling intervention was investigated in this study, the sustained effect and/or dose–response relationship for foam rolling intervention still is unclear. To clarify the sustained effect or dose–response relationship of foam rolling on eccentrically-damaged muscle, future studies are needed. Moreover, to investigate the effect of foam rolling on eccentrically-damaged muscle in the athletic population, further studies are needed, since the participants of this study were sedentary and nonathletes.

## 5. Conclusions

In conclusion, we investigated the effect of 90-s foam rolling intervention on eccentrically-damaged muscle. The study results indicate that muscle soreness and muscle function loss were improved, and the effect was greater in the subjects with greater muscle soreness and decreased muscle function by the ECC exercise. Therefore, foam rolling is an effective recovery tool for eccentrically-damaged muscles in sports and rehabilitation settings.

## Figures and Tables

**Table 1:** The changes in maximum voluntary isometric contraction (MVC-ISO) torque, maximum voluntary concentric contraction (MVC-CON) torque of knee extensors, and maximal voluntary range of motion (ROM) of passive knee flexion and muscle soreness at contraction, palpation, and stretching at baseline, before foam rolling intervention, and after foam rolling intervention.

	Baseline	Preintervention	Postintervention	F Value
MVC-ISO (Nm)	151.4 ± 22.8	96.8 ± 39.5 *	112.4 ± 30.0 *^,†^	22.6
MVC-CON (Nm)	147.1 ± 23.9	82.3 ± 30.7 *	100.3 ± 31.4 *^,†^	44.9
ROM (°)	145.6 ± 7.3	140.5 ± 6.3 *	149.2 ± 6.1 *^,†^	24.2
Muscle soreness				
At contraction (mm)	10.1 ± 8.6	25.3 ± 17.0 *	15.9 ± 12.9 ^†^	10.2
At stretching (mm)	31.8 ± 23.1	47.6 ± 22.6 *	25.1 ± 21.5 ^†^	13.5
At palpation (mm)	19.9 ± 14.4	44.1 ± 16.5 *	25.0 ± 13.6 ^†^	26.5

* A significantly (*p* < 0.05) different from the baseline value; ^†^ A significantly (*p* < 0.05) different from the preintervention value.

## Data Availability

All data generated or analyzed during this study are included in this published article.
